# Case Report: Suprasellar Pituitary Adenoma Presenting With Temporal Lobe Seizures

**DOI:** 10.3389/fsurg.2020.598138

**Published:** 2020-12-01

**Authors:** Christopher S. Hong, Ramana Gorrepati, Adam J. Kundishora, Aladine A. Elsamadicy, Patricia R. Peter, Eyiyemisi C. Damisah, R. Peter Manes, Sacit Bulent Omay

**Affiliations:** ^1^Department of Neurosurgery, Yale School of Medicine, New Haven, CT, United States; ^2^Section of Endocrinology, Department of Medicine, Yale School of Medicine, New Haven, CT, United States; ^3^Division of Otolaryngology, Department of Surgery, Yale School of Medicine, New Haven, CT, United States

**Keywords:** pituitary, macroadenoma, seizures, epilepsy, case report

## Abstract

Seizures in patients with pituitary pathology are uncommon and typically secondary to electrolyte disturbances. Rarely, seizures have been described from mass effect related to large prolactinomas undergoing medical treatment. We describe a 54 year-old male who presented with a first-time generalized seizure, secondary to a pituitary macroadenoma compressing the left temporal lobe. His seizures abated after endoscopic endonasal debulking of the tumor. This report highlights isolated seizures as a potential sole presenting symptom of pituitary macroadenomas without visual or endocrine dysfunction. Prompt surgical debulking to relieve mass effect on the temporal lobe may effectively prevent further seizure activity.

## Introduction

Pituitary adenomas are relatively common, benign tumors that are often diagnosed as an incidental finding. When symptoms occur, patients typically present with neurologic symptoms such as visual deficits and headaches from mass effect on the optic apparatus and/or endocrine dysfunction from hormonal secretion (1). In this report, we describe a unique case of a non-functioning pituitary adenoma in a patient with a history of previously undiagnosed focal aware seizures who presented with a new-onset secondarily generalized seizure but was otherwise asymptomatic. We describe the management of mass effect-related seizures from pituitary adenomas, based on our own experience and review of the literature.

## Case Description

**Figure 1:**
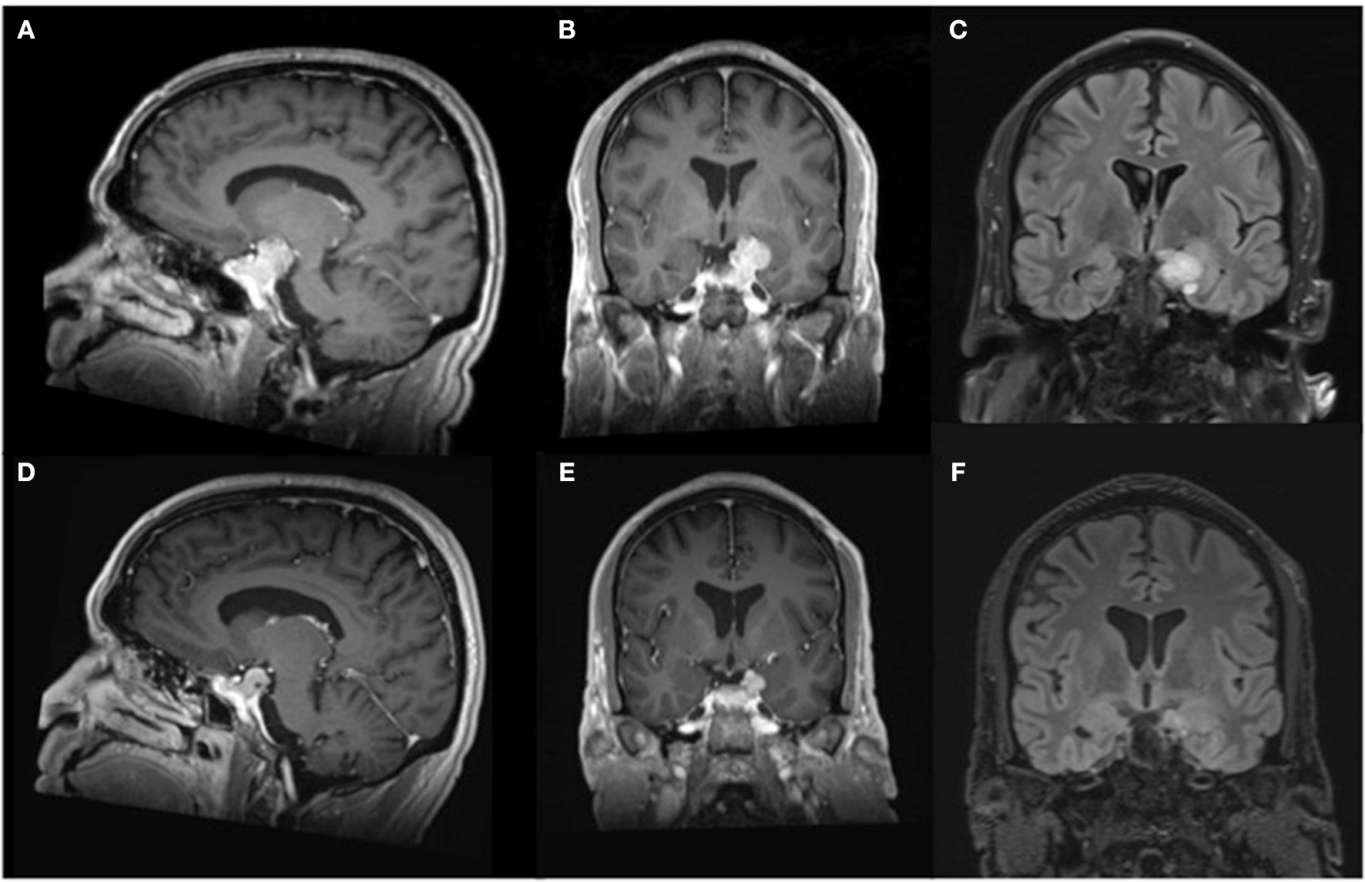
Magnetic resonance imaging of the brain. Representative **(A)** parasaggital and **(B)** coronal pre-operative T1-weighted imaging after contrast administration demonstrates an avidly enhancing multilobulated sellar lesion causing mass effect upon the optic apparatus, as well as notable displacement of the left uncus and hippocampal gyrus, resulting in compression of the medial temporal lobe. **(C)** Coronal pre-operative T2-weighted FLAIR shows mild parenchymal edema within the adjacent temporal lobe. Repeat imaging obtained 4 months after surgical decompression revealed decreased size of the enhancing lesion on **(D)** parasaggital and **(E)** coronal T1-weighted imaging after contrast administration with resultant diminished mass effect on the left medial temporal lobe and **(F)** absence of edema on T2-weighted FLAIR imaging.

A 54 year-old right-handed male with no significant past medical history presented to our emergency department after suffering a first-time generalized seizure at home. Upon further questioning, the seizure was preceded by a metallic taste sensation, followed by right arm shaking prior to generalization and loss of consciousness. He also endorsed infrequent episodic sensations of metallic taste in his mouth over the past 10 months that self-resolved after several minutes. Based on this history, his presentation was consistent with secondary generalization of baseline focal aware seizures. After initiation of anti-seizure therapy with levetiracetam, magnetic resonance imaging (MRI) of the brain was obtained, demonstrating a large sellar mass, extending into the left cavernous sinus and compressing the optic chiasm, as well as superiorly directed growth into the temporal fossa, abutting the left uncus and hippocampus, resulting in mild mass effect upon the medial left temporal lobe with associated parenchymal edema (Figures 1A–C). Further laboratory work-up revealed normal serum electrolytes and endocrine panel (Table 1). Additional electroencephalogram (EEG) monitoring was deferred in favor of proceeding directly to surgical resection via an endoscopic endonasal approach that facilitated resection of the sellar and suprasellar components and debulking of the tumor that extended toward the medial temporal lobe. Histopathology and immunohistochemical staining for hormonal markers revealed a WHO I grade non-functioning pituitary adenoma with a Ki-67 index <2%. Post-operative imaging obtained 2 weeks later revealed expected residual suprasellar tumor within the temporal fossa with complete decompression of the optic apparatus and reduced mass effect upon the temporal lobe. Repeat MRI obtained 4-months after surgery demonstrated stable residual tumor on the temporal lobe and resolution of parenchymal edema (Figures 1D–F). At 6-month follow-up, the patient remained free of focal aware and generalized seizures on continued anti-seizure therapy with plans to potentially wean treatment, pending elective EEG monitoring.

**Table 1:** Laboratory values obtained at time of presentation.

**Lab marker**	**Value**	**Range**
TSH	2.39	0.27–4.2 μLU/mL
fT4	0.8	0.8–1.5 ng/dL
FSH	8.2	2.5–10.2 mlU/mL
LH	5.2	1.7–8.6 mlU/mL
Cortisol 8 AM	10.1	6.0–18.4 μg/dL
PRL	7.5	4.0–15.2 ng/mL
Sodium	141	136–144 mmol/L
Potassium	3.8	3.3–5.1 mmol/L
Chloride	107	98–107 mmol/L
Glucose	118	70–100 mg/dL
Calcium	8.3	8.8–10.2 mg/dL
Magnesium	2.0	1.7–2.4 mg/dL

## Discussion

This is a unique case of isolated seizure presentation in an otherwise asymptomatic patient found to have a non-functioning pituitary macroadenoma causing compression of the medial temporal lobe. Development of seizures in patients with pituitary pathology is rare and typically secondary to severe electrolyte disturbances at time of presentation or in the post-operative period (2, 3). Otherwise, seizures in association with pituitary pathologies have been most commonly observed as a complication of medically treated large prolactinomas (4–6). Niwa et al. reported development of seizures in three of nine patients being treated with bromocriptine therapy for prolactinomas (4) and reported that MRI evidence of hemosiderin deposition on the medial temporal lobe after intratumoral hemorrhage during medical treatment for prolactinomas was the instigator for seizure development, a notion also reported by others (5).

Seizures have also been associated with large prolactinomas as a presenting symptom at time of diagnosis. Papanastasiou et al. described a patient who presented with new generalized tonic-clonic seizures secondary to a biochemically diagnosed large prolactinoma, which was successfully treated with cabergoline and anti-seizure therapy (7). Deepak et al. described six patients presenting with a history of seizures at time of diagnosis of prolactinomas, five of whom exhibited complex, partial seizures secondary to invasion of the medial temporal lobe and had been experiencing these symptoms for 2–23 years prior to diagnosis (8). The remaining patient presented with a first-time generalized tonic-clonic seizure secondary to suprasellar extension of the tumor reaching up into the third ventricle. After initiation of anti-dopaminergic treatment, all patients experienced a rapid reduction in seizure frequency. More recently, Shijo et al. described a patient with cranial nerve III-VI deficits and headaches secondary to pituitary apoplexy who suffered a secondary generalized seizure shortly after presentation (9). Biochemical testing was consistent with a non-functioning pituitary adenoma. Imaging showed that the tumor extended far laterally beyond the cavernous sinus with mass effect on the adjacent temporal lobe. After medical treatment of his seizures, the patient underwent resection of the tumor via an endoscopic endonasal approach, and post-operative imaging showed no evidence of recurrent tumor and absence of edema or hemosiderin along the temporal lobe. Although EEG showed no epileptiform activity 20 days after surgery, anti-seizure medications were continued at the patient's request, and the patient remained seizure-free at 1-year follow-up. The findings from the aforementioned studies are summarized in Table 2.

**Table 2:** Summarized review of the literature on seizures associated with pituitary adenomas.

**References**	**Number of patients**	**Pituitary tumor pathology**	**Clinical presentation**	**Imaging features**	**Seizure treatment**	**Clinical outcome**
Niwa et al. (4)	3	Prolactinoma	Seizures developed in tumors undergoing treatment with bromocriptine treatment	Tumors had lateral extension involving cavernous sinus Hemosiderin deposits seen on medial surface of temporal lobe	NS	NS
Hashizume et al. (5)	1	Prolactinoma	Seizures developed in a patient on chronic bromocriptine treatment for giant prolactinoma	Hemosiderin deposit seen on left medial temporal lobe	L anterior temporal lobectomy, partial hippocampectomy	Seizure free for 8 months
Deepak et al. (8)	6	Prolactinoma	History of seizures (median 2 years) preceding or at time of tumor diagnosis	Tumor extension to medial temporal lobe in 5/6 patients	Anti-seizure therapy (phenytoin, cabamazepine) in 5/6 patients, anti-dopamingeric treatment in all patients	4 patients seizure free (range: 18 months to 15 years), remaining 2 patients with reduced seizure frequency
Dhanwal and Sharma (6)	1	Prolactinoma	Seizures and visual loss developed after 6 months of cabergoline treatment for giant prolactinoma	Optic chiasmal and frontal lobe herniation into the sella	Phenytoin, cabergoline dose reduction (for visual loss)	Seizure free, marginal visual improvement (follow-up time period NS)
Papanastasiou et al. (7)	1	Prolactinoma	Seizures and visual loss developed at time of presentation/diagnosis of giant prolactinoma	Giant prolactinoma with suprasellar and lateral cavernous sinus extension	Anti-seizure therapy (NS) with no seizure recurrence	Seizure free (follow-up time period NS) experienced worsening vision from brain/chiasmal herniation into sella during cabergoline therapy, requiring craniotomy
Shijo et al. (9)	1	NS	Acute onset seizure, cranial nerve palsies (III–VI), and headache	Tumor extension into suprasellar region and cavernous sinus	Levetiracetam, surgery with pathology consistent with pituitary apoplexy	Seizure-free for 12 months
Index case	1	Non-functioning pituitary adenoma	New onset generalized seizure at time of diagnosis	Tumor extension into cavernous sinus, medial temporal lobe	Levetiracetam, surgery	Seizure-free for 6 months

*NS, not specified*.

Unlike most of the prior studies describing seizures from pituitary adenomas, biochemical testing of our patient at time of presentation did not reveal underlying hormonal abnormalities and therefore, medical therapy with anti-dopaminergic therapy was not an option. As such, surgical intervention via an endoscopic endonasal approach was pursued with the primary goals of relieving mass effect on the temporal lobe to alleviate further seizures and to achieve a pathologic diagnosis. Given the patient was otherwise neurologically intact besides seizure presentation and the laterality of the tumor toward the dominant hemisphere, the endoscopic endonasal approach was favored over an open craniotomy that carried greater risk for neurologic co-morbidity. Unlike the case of Shijo et al. (9) the temporal lobe involvement in our patient's tumor was more superior rather than directly lateral, thus explaining a lack of cranial nerve deficits. Residual tumor on the temporal lobe was expected with an endoscopic endonasal approach, considering the limitations of endoscopic endonasal visualization beyond the immediate suprasellar region into the temporal fossa. Given the final pathologic diagnosis demonstrated a benign, WHO I pituitary adenoma with a low Ki-67 index <2%, we chose to monitor the residual tumor with surveillance imaging, rather than pursue further surgery via a transcranial approach, which has been described as a primary or staged approach for complex, giant pituitary macroadenomas in patients, typically with pre-existing visual and/or hormonal deficits (10–12). Surveillance imaging of our patient has not revealed tumor growth, but if this were to occur, we would pursue stereotactic radiosurgery as the least invasive option to the patient, a strategy that has previously been reported to result in durable local tumor control (13), prior to considering further options such as a second surgery via an open approach. At last follow-up, our patient continues on anti-seizure treatment, seizure and aura-free, and with plans to taper therapy pending elective EEG monitoring, which we believe is possible given the relief of mass effect on the temporal lobe after tumor debulking. In contrast, based on the existing literature (4, 5), in cases where intratumoral hemorrhage occurs with subsequent hemosiderin deposition on the temporal lobe, indefinite anti-seizure therapy may be indicated for seizure control, and consideration of further surgical options may be necessary should medical treatment of seizures fail.

The majority of the literature has described seizure presentation from pituitary adenomas after medical therapy for macroprolactinomas. Our patient, however, is a unique case of a non-functioning pituitary adenoma causing seizures as the sole presenting symptom at time of diagnosis. Accurate biochemical testing is imperative in these patients, as prolactinomas may be medically treated with anti-dopimanergic and anti-seizure therapies. In contrast, for patients with biochemical testing suggestive of a non-functioning pituitary adenoma, surgical intervention should be aimed at decompression of the epileptogenic focus, which typically involves the ipsilateral temporal lobe. In cases where significant intratumoral hemorrhage is present, in which hemosiderin deposition may perpetuate seizure activity, prolonged or indefinite anti-seizure therapy may be necessary if gross total resection of the tumor cannot be achieved.

## Data Availability Statement

The original contributions presented in the study are included in the article/supplementary material, further inquiries can be directed to the corresponding author/s.

## Ethics Statement

Ethical review and approval was not required for the study on human participants in accordance with the local legislation and institutional requirements. The patients/participants provided their written informed consent to participate in this study.

## Author Contributions

CH, RG, AK, and AE drafted the manuscript. PP, ED, RM, and SO edited the manuscript. All authors were involved in the clinical care of the patient and approved the final version of the manuscript at time of submission.

## Conflict of Interest

The authors declare that the research was conducted in the absence of any commercial or financial relationships that could be construed as a potential conflict of interest.
